# Measuring the Transition Rates of Coalescence Events during Double Phase Separation in Microgravity

**DOI:** 10.3390/molecules22071125

**Published:** 2017-07-06

**Authors:** Ana Oprisan, Yves Garrabos, Carole Lecoutre, Daniel Beysens

**Affiliations:** 1 Department of Physics and Astronomy, College of Charleston, Charleston, SC 29424, USA; 2 CNRS, Institut de Chimie de la Matière Condensée de Bordeaux, ESEME, Université de Bordeaux, UPR 9048, F-33600 Pessac, France; yves.garrabos@icmcb.cnrs.fr (Y.G.); carole.lecoutre@icmcb.cnrs.fr (C.L.); 3 Physique et Mécanique des Milieux Hétérogènes, UMR 7636 CNRS-ESPCI-Université Pierre et Marie Curie - Université Paris Diderot, 10 rue Vauquelin, 75005 Paris, France; daniel.beysens@espci.fr

**Keywords:** phase separation, microgravity, binary coalescence

## Abstract

Phase transition is a ubiquitous phenomenon in nature, science and technology. In general, the phase separation from a homogeneous phase depends on the depth of the temperature quench into the two-phase region. Earth’s gravity masks the details of phase separation phenomena, which is why experiments were performed under weightlessness. Under such conditions, the pure fluid sulphur hexafluoride (SF${}_{6}$) near its critical point also benefits from the universality of phase separation behavior and critical slowing down of dynamics. Initially, the fluid was slightly below its critical temperature with the liquid matrix separated from the vapor phase. A 0.2 mK temperature quench further cooled down the fluid and produced a double phase separation with liquid droplets inside the vapor phase and vapor bubbles inside the liquid matrix, respectively. The liquid droplets and the vapor bubbles respective distributions were well fitted by a lognormal function. The evolution of discrete bins of different radii allowed the derivation of the transition rates for coalescence processes. Based on the largest transition rates, two main coalescence mechanisms were identified: (1) asymmetric coalescences between one small droplet of about 20 μm and a wide range of larger droplets; and (2) symmetric coalescences between droplets of large and similar radii. Both mechanisms lead to a continuous decline of the fraction of small radii droplets and an increase in the fraction of the large radii droplets. Similar coalescence mechanisms were observed for vapor bubbles. However, the mean radii of liquid droplets exhibits a $t^{1/3}$ evolution, whereas the mean radii of the vapor bubbles exhibit a $t^{1/2}$ evolution.

## 1. Introduction

Phase transition is a ubiquitous phenomenon in nature (e.g., the water cycle), science and technology [1]. In general, the phase separation from a homogeneous phase depends on the depth of the temperature quench into the two-phase region. The phase separation could occur through spinodal decomposition [2,3,4,5,6,7,8,9,10] or nucleation and growth [3,10,11,12,13,14,15,16]. These two processes can result in quite different morphologies. Usually, during the initial stage of the spinodal decomposition, we observe bicontinuous structures, whereas, during the nucleation and growth, we observe isolated domains in another continuous phase. The above classical theories of phase separation have some limitations. For example, the classical nucleation theory describes quite well the condensation of supercooled vapors, but gives only qualitative results when applied to supersaturated fluids. As a result, the nucleation and growth theory was further augmented—for example, with the density functional theory [17,18,19] and the diffuse interface theory [20]. Spinodal decomposition or nucleation path also depends on the volume fraction of the minority phase [9,21]. Additionally, the presence of solid walls and the wetting effects dramatically modify phase separation dynamics [22,23,24,25,26,27].

From an experimental perspective, the method most frequently used for producing phase separation is based on temperature quench in which a sample in a homogeneous state is usually heated up or cooled down. However, due to the short time scale of the phase separation and the finite quench speed, it is very difficult to study phase separation kinetics. Additionally, Earth’s gravity hampers experimental observation of fast phase separation because the vapor bubbles rise up and liquid droplets fall down, which quickly creates a flat meniscus determined by the gravity-induced coalescence of bubbles or droplets [28,29]. The microgravity environment for these experiments suppress the gravitational coalescence and allow a reasonably long time of observation of the phase-separating liquid droplets. In addition, dealing with fluids near their critical point allows critical scaling universality to be used to generalize the results to all fluids, and critical slowing down of the phase separation dynamic allows for the observation of phenomena that would otherwise be undetected due to their very fast dynamics. The DECLIC (Dispositif pour l’Étude de la Croissance et des Liquides Critiques) flight model facility is a multi-user facility for investigating critical fluids behavior and directional solidification of transparent alloys. The compact design contains three inserts, of which we refer here only to the ALI (Alice Like Insert) dedicated to the study of sulphur hexafluoride (SF${}_{6}$) as a near-ambient temperature critical fluid. The program covers a whole characterization of SF${}_{6}$, ranging from thermodynamic quantities measurements (thermal diffusivity, heat capacity and turbidity near the critical point) to boiling effects studies [30].

This study reports on the double phase separation process that takes place after a supercritical SF${}_{6}$ fluid is brought from an initial state, which is already in a two-phase state slightly below the critical temperature, to a temperature 0.2 mK below the initial value thanks to a temperature quench. Although the one-step phase separation process has been studied by several authors in weightlessness [11,12,13,24,28,31,32,33,34,35,36,37,38,39,40], double quench has only been the object of very few studies [35,41].

As discussed in detail in [41], the 0.2 mK temperature quench produced further phase separation with (1) vapor bubbles inside the majority liquid phase (the latter wetting the walls of the sample cell unit); and (2) liquid droplets inside the initially separated vapor phase. The novelty of this study is the quantitative investigation of the liquid droplets and vapor bubbles distributions, respectively, observed during the double phase separation process. We derived for the first time, to our knowledge, the transition rates that describe the evolution of different radii bins from recorded images of phase-separating systems. Two dominant coalescence mechanisms were found: (1) an asymmetric coalescence between small liquid droplets and a relatively wide range of other droplet sizes; and (2) a symmetric coalescence between large droplets of similar sizes.

## 2. Experimental Setup

**Figure 1:**
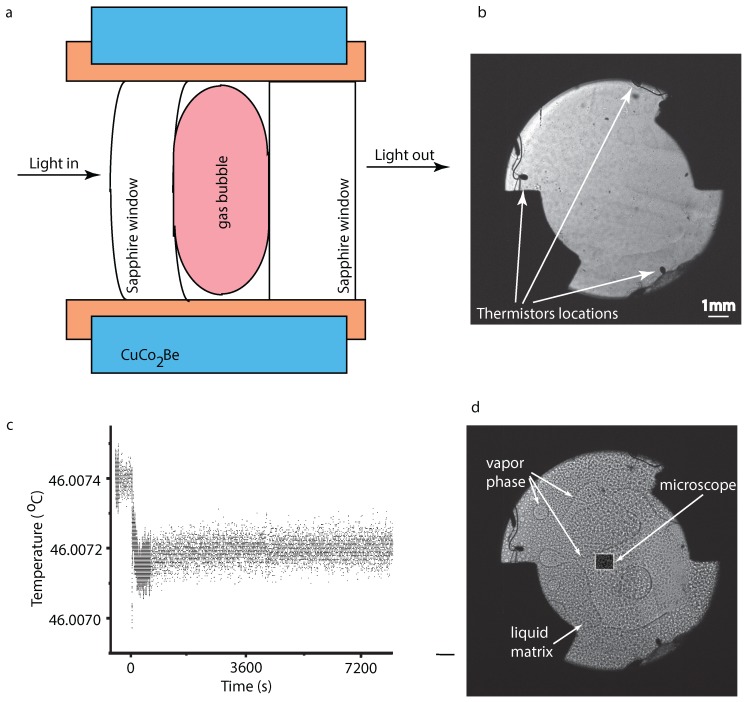
(**a**) a schematic cross section (not to scale) of the Direct Observation Cell (DOC) dedicated to studying sulfur hexafluoride (SF${}_{6}$) phase separation in Dispositif pour l’Étude de la Croissance et des Liquides Critiques (DECLIC) with Alice Like Instrumentation (ALI). experiments; (**b**) a wide field of view (WFOV) image of the DOC at equilibrium in the two-phase range recorded 100 s prior to the application of 0.2 mK temperature quench; (**c**) the DOC was slightly below the critical temperature when the cooling down temperature quench of 0.2 mK was applied at *t* = 0 s; (**d**) as a result of the 0.2 mK thermal quench, a double phase separation takes place with liquid droplets nucleating inside the vapor phase and vapor bubbles forming inside the liquid matrix. A microscope magnifies a 1 mm × 1 mm square area at the center of the DOC.

The DECLIC experimental setup was described extensively in previous publications [30,41,42]. DECLIC used Alice Like Instrumentation (ALI) to carry out phase separation experiments near the critical point of SF${}_{6}$ at room temperature in the weightlessness. The optical pressurized cells (or the Direct Observation Cell—DOC) contained a heater device as a transparent resistive layer appropriate for light transmission observation. The DOC was filled with SF${}_{6}$ at its vapor-liquid critical point $T_{c}=318.737$ K, (45.587 ${}^{\circ }$C), p=3.73 MPa, and ρ=742.6 kg m${}^{-3}$. A sketch of the cell is shown in Figure 1a (see also [41,43,44] for more details). The cylindrical DOC had an inner diameter of *D* = 10.6 mm and inner thickness of *e* = 4.115 mm, and was prepared with a mean density $\rho =\rho_{c}+2\%$. Three small thermistors were located inside the fluid volume, so that three local temperatures are measured close to the vapor–liquid interface in the microgravity environment (see Figure 1b). The light source was a 633 nm He-Ne laser.

The optical system allowed interlaced recordings of both Wide Field Of View (WFOV) images, which covers a circular area with diameter of 10.6 mm, and Narrow Field Of View (NFOV) images, which only covers a 1 mm × 1 mm area located at the center of the DOC [41,42]. Starting from an initial state, which is slightly below the critical temperature where the system is already phase-separated as shown in Figure 1b, the DOC is further cooled down by a temperature quench of 0.2 mK (see Figure 1c) that allowed the supercritical fluid to cross into a double phase separation regime in which inside the vapor bubbles we observed liquid droplets and inside the liquid matrix there were vapor bubbles (see Figure 1d).

All data analyzed here belong to ALI sequence 7 for which the temperature quench started at t=0 s, i.e., the image reference index of $img_{ref}$ = 1,123,710,576 with Δ = 23 frames per second. For example, the image shown in Figure 1b has an index of img = 1,123,708,278, which means it was taken at the actual time $t=(img-img_{ref})/\Delta$ = (1,123,708,278 − 1,123,710,576)/23 = −99.9 s from the temperature quench, i.e., approximately 100 s prior to 0.2 mK temperature quench. The image index for the snapshot shown in Figure 1d was img = 1,123,782,720, which corresponds to about 3136.7 s after the thermal quench.

## 3. Narrow Field of View (NFOV) Image Processing

Macroscopic images, or WFOV images, such as those shown in Figure 1b,d, are interlaced with microscopic images, or NFOV images, taken with a microscope objective with a magnification of ×12 (see Figure 1d for microscope location). In the NFOV images, one pixel is equivalent to 0.977 μm.

**Figure 2:**
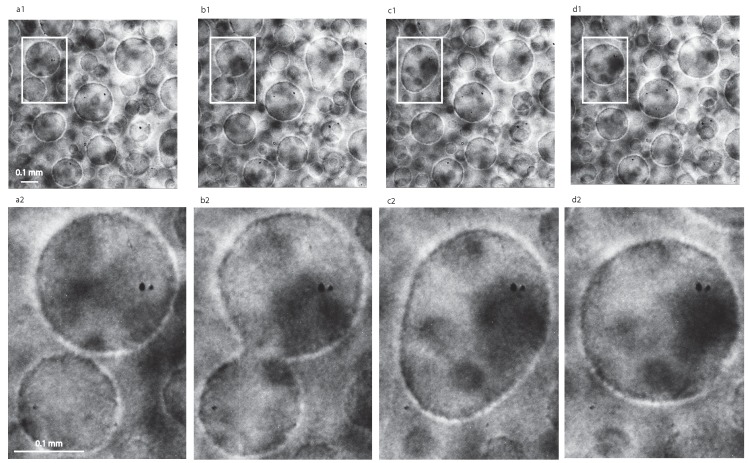
Binary collisions between vapor bubbles in narrow filed of view (NFOV) images at successive times (**a**) 6607.3 s; (**b**) 6879.7 s; (**c**) 6970.4 s; and (**d**) 7061.2 s. The highlighted white-bordered rectangle in panes (**a1**–**d1**) show two vapor bubbles of slightly different radii that approached each other (**a1**,**a2**) and then form a continuous bubble (**b1**,**b2**), which slowly changes into an oblongated shape (**c1**,**c2**) and finally becomes spherical again (**d1**,**d2**). We also magnified the region of interest for better visualization of coalescence (see panels **a2**–**d2**).

Initially, the microscope captured images from inside a large vapor bubble that phase-separated before we applied the 0.2 mK temperature quench (as seen in Figure 1d and labeled “vapor phase”, i.e., prior to applying the 0.2 mK temperature quench). Inside this large vapor bubble, we observed coalescences between liquid droplets. As the time passes, the vapor phase recedes and the large vapor bubble seen on the right side of Figure 1d becomes smaller and smaller. As a result, at later stages, the microscope captured images from the region marked “matrix liquid” phase in Figure 1d. In this region, we also noticed coalescences as seen in Figure 2, but they take place between the vapor bubbles embedded in the liquid majority phase.

The high resolution recording and the slowing down of coalescence processes due to microgravity conditions allowed us to observe binary collisions as shown in Figure 2. Due to microgravity slowing down of all critical processes, we can follow a coalescence between two vapor bubbles for more than seven minutes (see Figure 2). Although we only focused and magnified the coalescence in the upper left corner of Figure 2a1–d1, there are other binary coalescences visible (notice the one in the upper right corner of Figure 2).

## 4. Results

### 4.1. Liquid Droplets Dynamics from NFOV Images

Inside any large vapor bubbles formed during the initial phase separation process prior to the application of the 0.2 mK thermal quench, a dynamic process of continuous condensation of liquid droplets from supercritical phase takes place. As we noticed from the WFOV images, the large vapor bubbles are in continuous and slow motion due to internal processes, such as droplets nucleation and expansion of the wetting layer at cell boundaries, Brownian motion, g-jitter, and residual steady gravity due to the fact that the sample is not at the spacecraft center of mass. As a result of the macroscopic motion, some microscopic recordings caught the slow drift of the interface between a large vapor bubble (with liquid droplets inside) and the majority liquid phase (with vapor bubbles inside).

**Figure 3:**
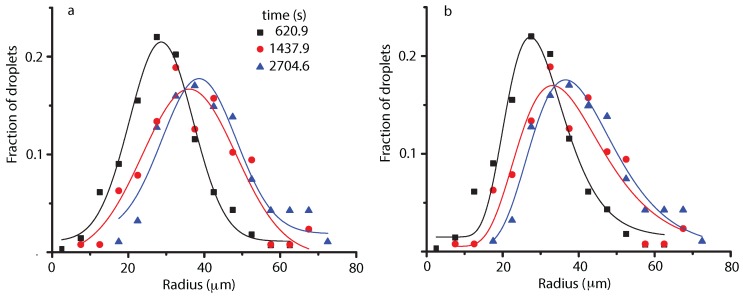
Histograms of microscopic views at successive times 620.9 s (solid squares), 1437.9 s (solid circles), and 2704.6 s (solid triangles). The continuous lines show the corresponding Gauss (**a**) and lognormal (**b**) fits. For image at 620.9 s (solid squares) the center of Gauss distribution is at $x_{cG}$ = (28.7 ± 0.4) μm and of lognormal is at $x_{cLN}$ = (29.5 ± 0.8) μm. The corresponding standard deviations are $w_{G}$ = (8.4 ± 0.5) μm and $w_{LN}$ = (0.28 ± 0.03) μm. For the snapshot at 1437.9 s (solid circles), $x_{cG}$ = (36.1 ± 1.1) μm and $x_{cLN}$ = (37.0 ± 1.8) μm with the standard deviations and $w_{G}$ = (12.3 ± 2.4) μm and $w_{LN}$ = (0.33 ± 0.06) μm. For the image at 2704.6 s (solid triangles), $x_{cG}$ = (38.6 ± 1.0) μm and $x_{cLN}$ = (39.9 ± 1.0) μm with $w_{G}$ = (9.8 ± 1.5) μm and $w_{LN}$ = (0.29 ± 0.04) μm.

We fitted the NFOV liquid droplets distributions both to Gauss (Figure 3a) and lognormal (Figure 3b) functions to gauge possible trends over time. We found out that there is no significant difference in the goodness of fit between the two fitting functions. However, we favor the lognormal distribution (Figure 3b) due to the presence of the asymmetric, long tail in the experimental data (see also [41] for details). Some radii bins, e.g., around 60 μm, are significantly lower than the theoretical curves shown in Figure 3. One reason could be the fact that the 1 mm × 1 mm microscope window simply did not capture correctly the 60 μm radii bins at that particular time. We assumed that the distribution of droplets as recorded by the 1 mm × 1 mm microscope is representative for the entire bulk distribution. However, the homogeneity assumption may not be locally valid and fluctuations are possible, especially since the system is still evolving through coalescences. Another contributing factor is the random coalescence processes that may at some particular instances deplete the droplets distributions with some specific radii, e.g., 60 μm, while generating larger droplets.

**Figure 4:**
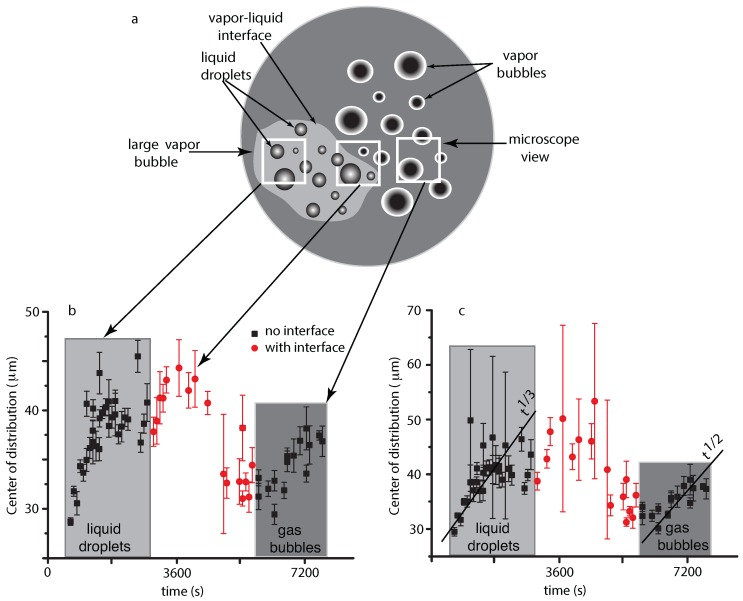
(**a**) a schematic representation of the sample cell unit (not at scale) containing one large vapor bubble inside the liquid matrix. The microscope view shifts over time and at early stages captures liquid droplets distributions from inside the large vapor bubble (see the leftmost white-bordered square). Later on, the microscope captures the vapor–liquid interface and a mixture of distributions (see the middle white-bordered square). At late stages, the microscope view only captures vapor bubbles embedded in the liquid matrix (see the rightmost white-bordered square). The location of the center of the Gaussian (**b**), respectively, lognormal (**c**) distributions shift over time. At first, the distribution of liquid droplets shifts towards larger radii (solid squares inside the grayed “liquid droplets” area). At some point, the interface line between the large vapor bubble and the liquid matrix drifts into focus between 2981.2 s and 5790.3 s (solid red circles) and significantly changes the trend of the distributions due to boundary effects. While the best fit is still a lognormal distribution, the center of the distribution seems to remain constant and even moves towards smaller radii. At a later time, after the vapor bubble completely receded from focus, the distribution of liquid droplets also shows an increase in its mean radius (see the grayed rectangle marked “liquid droplets”). The continuous lines in (**c**) indicate a $t^{1/3}$ evolution for liquid droplet radii and a $t^{1/2}$ evolution for vapor droplets (see [41] for data fitting details and a discussion regarding possible coalescence mechanisms).

The most noticeable result regarding droplet radii distribution is that initially the center of the distribution shifts towards larger and larger values (see Figure 3). This is consistent with the Brownian coalescence mechanism and the radii increase as $t^{1/3}$ (see the continuous line inside the first grayed rectangle in Figure 4c, and, for fitting details, see [41]). After the initial fast increase in the average radii, the distributions flattened, which suggests that the process reached a steady state (see the solid red circles in the middle portion of Figure 4b,c). Initially, the microscope captured images of liquid droplets inside the large vapor bubble seen in Figure 1d and schematically represented by the leftmost white-bordered square in Figure 4a. The mean radius of the distribution of liquid droplets initially shifts towards larger radii (see the grayed rectangles marked “liquid droplets” in Figure 4b,c).

At later times, we notice that the mean radius remains almost constant. While the large vapor bubble recedes, a phase separation interface between the liquid matrix and the vapor bubble comes into focus as shown schematically in the middle white-bordered square of Figure 4a and the corresponding solid red circles in Figure 4b,c. In this range of durations, the distributions are skewed due to the boundary effects induced by the moving interface line. Eventually, the vapor bubble recedes such that the interface is no longer visible in NFOV images, as shown in the rightmost white-bordered square in Figure 4a, and we only record vapor bubbles inside the liquid matrix (see the grayed rectangle marked “vapor bubbles” in Figure 4b,c). In this region, we found that the mean radius of vapor bubbles increases due to coalescences with $t^{1/2}$ (see the continuous line inside the last grayed rectangle in Figure 4c and also [41] for detailed discussions of possible mechanisms).

Although the mean radius of the distributions may increase over time because of coalescences, a more detailed picture of the coalescence mechanism emerged from the evolution of different radii bins. For this purpose, we binned all NFOV distributions with 10 pixels (≈10 μm). When we focus on the initial stage of coalescence in the NFOV, i.e., for times below 1800 s and the corresponding $t^{1/3}$ trend in Figure 4c, it seems that the relative fraction of the droplets of radii below 40 μm follow a descendent trend. At the same time, the relative fraction of the droplets with radii larger than 50 μm seem to have an ascendant path (see [41] for detailed a description). This evolution could be the result of coalescence events that deplete the distribution of small droplets in favor of generating larger droplets (see the solid squares inside the first grayed rectangle of Figure 4b,c).

### 4.2. Vapor Bubbles Dynamics from NFOV Images

Inside the liquid matrix, a dynamic process of phase separation of vapor bubbles from supercritical phase takes place. As in the previous subsection, we also fitted the NFOV vapor bubbles distributions both to Gauss and lognormal functions to gauge possible trends over time (not shown, but see Figure 3 for similar trends seen in liquid droplets case and also [41]). Both the Gaussian and lognormal fit of the vapor bubbles distributions show similar trends. While the large vapor bubble (with liquid droplets inside) receded from the field of view, we initially recorded vapor bubbles distributions in the presence of a moving phase separation interface for times below approximately 5000 s. As we discussed in the previous subsection, such distributions (marked with solid circles in Figure 4b,c) are skewed due to the finite size effects near the phase separation interface. For this reason, we did not consider them when analyzing the trend of the mean radius versus time (see the continuous lines in Figure 4c that show the $t^{1/3}$ power law for liquid droplets evolution and $t^{1/2}$ power law for vapor bubbles evolution, respectively. For fitting details see also [41]).

After the phase separation interface is out of the field of view, we recorded images from the bulk of the liquid matrix that contained vapor bubbles undergoing coalescences. The most noticeable result regarding vapor bubbles radii distribution is that the center of the distribution shifts towards larger and larger values (see the second grayed rectangle in Figure 4b,c). This is consistent with a faster than Brownian coalescence mechanism, possibly a directional motion of vapor bubbles due to a composition Marangoni force [45], possible g-jitter and remaining steady gravity due to the fact that the sample is not at the spacecraft center of mass [41], and the radii increase as $t^{1/2}$ (see the continuous line inside the second grayed rectangle in Figure 4c).

As in the preceding subsection, a more detailed picture of the coalescence mechanism emerges when we investigate the evolution of different radii bins. During the initial stage of coalescences in the NFOV (for times below 5000 s), it seems that the dynamic is similar to the one observed across the phase separation interface inside the large vapor bubble. During the ascending trend of the average radius (see the grayed rectangles marked “vapor bubbles” in Figure 4b,c) the fraction of the small droplets below 30 μm seem to decay over time and slightly increase for intermediate 40–50 μm bubbles. Larger bubbles with radii from 60 to 90 μm seem to also decrease their relative fraction, whereas large droplets over 100 μm increase their relative contribution to the distributions. This evolution could be the result of coalescence events and has different dynamics than previous measurements in liquid droplets (see the previous subsection).

## 5. Theoretical Modeling

**Figure 5:**
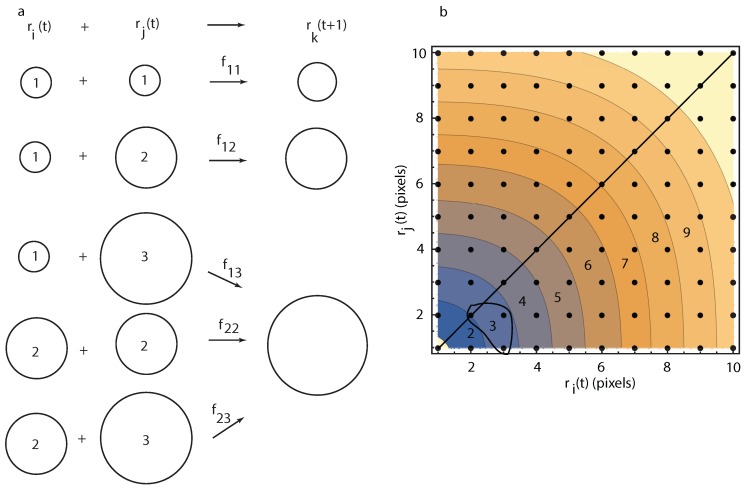
(**a**) binary collisions between droplets of radii $r_{i}\left(t\right)$ and $r_{j}\left(t\right)$ lead to a larger droplet of radius $r_{k}\left(t+1\right)$ with a collision transition rate $f_{ij}$. As the resultant droplet radius (binned in increments of one pixel) increased, the multiplicity of possible combinations increases. The number inside the circles symbolizing droplets represents their radii (in pixels) before coalescence; (**b**) the two-dimensional state space of the system at time step *t* shows all possible pairs of liquid droplet radii $r_{i}\left(t\right)$ and $r_{j}\left(t\right)<r_{i}\left(t\right)$ (below continuous diagonal line) that could lead to a new droplet of radius $r_{k}$ at iteration t+1. Each shaded contour plot covers a 1-pixel bin. For example, the shaded contour label with three pixels shows that it is the result of three possible types of coalescence events between droplets of following sizes: two pixels and two pixels, or one pixel and three pixels, or two pixels and three pixels.

From a mathematical perspective, if we assume that a new droplet can only occur due to a binary coalescence between two droplets of radii $r_{i}\left(t\right)$ and $r_{j}\left(t\right)$ then the resultant droplet has a radius $r_{k}\left(t+1\right)={\left(r_{i}{\left(t\right)}^{3}+r_{j}{\left(t\right)}^{3}\right)}^{1/3}$ as in Figure 5a. Since we are limited by the image resolution, it is natural to consider only radii with integer values in pixels. In the following, we consider that measured radii are binned with a 1-pixel (≈0.977 μm) increment. Therefore, by a coalescence event between a droplet of radius $r_{i}\left(t\right)$ = 1 pixel and $r_{j}\left(t\right)$ = 1 pixel it results in a droplet of radius $r_{k}\left(t+1\right)=1.26$ pixels, which will be counted in the 1-pixel bin at the time step t+1 (see Figure 5a). Such an event occurs with a transition rate $f_{11}$. Similarly, the droplets in the bin of size 2 pixels at time step t+1 could only be created by collisions between $r_{i}\left(t\right)$ = 1 pixel and $r_{j}\left(t\right)$ = 2 pixels (see Figure 5a). The transition rate for such a collision is $f_{12}$. The bin $r_{3}\left(t+1\right)$ could be generated by binary collisions $r_{i}\left(t\right)=1$ pixel and $r_{j}\left(t\right)$ = 3 pixels, or $r_{j}\left(t\right)=2$ pixels and $r_{j}\left(t\right)$ = 3 pixels, or $r_{i}\left(t\right)$ = 2 pixels and $r_{j}\left(t\right)$ = 2 pixels (see Figure 5a). Each possible combination has it own collision transition rate, i.e., $f_{13}$, $f_{23}$, and $f_{22}$, respectively. Similarly, the 4-pixel bin could be generated by binary collisions $r_{i}\left(t\right)$ = 1 pixel and $r_{j}\left(t\right)$ = 4 pixels, or $r_{i}\left(t\right)$ = 2 pixels and $r_{j}\left(t\right)$ = 4 pixels, $r_{i}\left(t\right)$ = 3 pixels and $r_{j}\left(t\right)$ = 4 pixels, or $r_{i}\left(t\right)$ = 3 pixels and $r_{j}\left(t\right)$ = 3 pixels, and so forth.

As a reminder, 1 pixel${}_{WFOV}\approx$ 12.04 μm in WFOV images and 1 pixel${}_{NFOV}\approx$ 0.977 μm in NFOV images. The two-dimensional state space of all possible binary events that generate a droplet of radius $r_{k}$ at iteration time t+1 is determined by all possible pairs as shown in Figure 5. The iteration time step is equal to the image acquisition sampling rate. The contour lines in Figure 5 separate points in integer value (pixel) radii. For example, the first contour line in Figure 5 goes between 0.5 and 1.5 pixels and only includes one possible data point, i.e., $r_{i}\left(t\right)=1$ pixel and $r_{j}\left(t\right)=1$ pixel, which could generate a droplet of radius ${\left(1^{3}+1^{3}\right)}^{1/3}=1.26$ pixels that rounds to a radius of 1 pixel at time t+1.

Similarly, the 3-pixel bin (with radii between 2.5 and 3.5 pixels) marked with a “3” inside the shaded area of Figure 5b, can only result from the following integer radii combinations: $r_{i}\left(t\right)=2$ pixels and $r_{j}\left(t\right)=2$ pixels, which gives $r_{k}\left(t+1\right)={\left(2^{3}+2^{3}\right)}^{1/3}\approx 2.52$ pixels, or $r_{i}\left(t\right)=1$ pixel and $r_{j}\left(t\right)=3$ pixels, which gives $r_{k}\left(t+1\right)={\left(1^{3}+3^{3}\right)}^{1/3}\approx 3.04$ pixels, or $r_{i}\left(t\right)=2$ pixels and $r_{j}\left(t\right)=3$ pixels, which gives $r_{k}\left(t+1\right)={\left(2^{3}+3^{3}\right)}^{1/3}\approx 3.27$ pixels. All of the above three possible integer radii coalescence events would be counted in the 3-pixel bin at time step t+1.

To summarize, the following recursive relationships describe the evolution of the number $n_{i}\left(t\right)$ of droplet with the radius $r_{i}\left(t\right)$ by accounting for all possible binary collisions that could generate such a droplet:

(1) $$\begin{array}{ccc} \Delta n_{1}\left(t\right) & = & f_{11}n_{1}{\left(t\right)}^{2},\\ \Delta n_{2}\left(t\right) & = & f_{12}n_{1}\left(t\right)n_{2}\left(t\right),\\ \Delta n_{3}\left(t\right) & = & f_{13}n_{1}\left(t\right)n_{3}\left(t\right)+f_{22}n_{2}{\left(t\right)}^{2}+f_{23}n_{2}\left(t\right)n_{3}\left(t\right),\\ \Delta n_{4}\left(t\right) & = & f_{14}n_{1}\left(t\right)n_{4}\left(t\right)+f_{24}n_{2}\left(t\right)n_{4}\left(t\right)+f_{34}n_{3}\left(t\right)n_{4}\left(t\right)+f_{33}n_{3}{\left(t\right)}^{2},\\ \Delta n_{5}\left(t\right) & = & f_{15}n_{1}\left(t\right)n_{5}\left(t\right)+f_{25}n_{2}\left(t\right)n_{5}\left(t\right)+f_{35}n_{3}\left(t\right)n_{5}\left(t\right)+f_{45}n_{4}\left(t\right)n_{5}\left(t\right)+f_{44}n_{4}{\left(t\right)}^{2},\\ \ldots & & \end{array}$$

where $\Delta n_{i}\left(t\right)=n_{i}\left(t+1\right)-n_{i}\left(t\right)$ is the finite difference increase in the relative frequency distribution of droplets of radius $r_{i}=i$ pixels during one time step, $f_{ij}$ represents the transition rates of a stable collision process between droplets of radii $r_{i}=i$ pixels and $r_{j}=j$ pixels with i≤j. To avoid cluttering the recursive formulas above, we only mention that $n_{6}\left(t+1\right)$ is determined by the set of transition rates $f_{16},f_{26},f_{36},f_{46},f_{45},f_{55}$; $n_{7}\left(t+1\right)$ is determined by the set $f_{17},f_{27},f_{37},f_{47},f_{56}$; $n_{8}\left(t+1\right)$ is determined by the set $f_{18},f_{28},f_{38},f_{48},f_{56},f_{57},f_{66}$; $n_{9}\left(t+1\right)$ is determined by the set $f_{19},f_{29},f_{39},f_{49},f_{59},f_{58},f_{68},f_{78},f_{77}$; etc.

Although for each possible cluster of size $r_{k}$ there are multiple possible binary collisions, e.g., a droplet of radius four pixels could be generated by four distinct binary collision events according to Equation (1), we could always select the largest (most probable) value of all $f_{ij}$ values that could generate a droplet of radius $r_{k}$. By connecting the states with the most likely (largest) binary collisions transition rates for each cluster shown in Figure 5, we obtained the phase space path of the system, which depends on many parameters, such as temperature, depth of the thermal quench that led to phase separation, volume fraction, etc.

We notice from Equation (1) that the transition rates $f_{11}\left(t\right)=\left(n_{1}\left(t+1\right)-n_{1}\left(t\right)\right)/n_{1}^{2}\left(t\right)$ and $f_{12}\left(t\right)=\left(n_{2}\left(t+1\right)-n_{2}\left(t\right)\right)/\left(n_{1}\left(t\right)n_{2}\left(t\right)\right)$ are first order recursions as they only involve current state time *t* and the immediate next state at time t+1. However, for larger radii $r_{k}\geq 2$ (in pixels), there is a multiplicity of possible ways of generating a droplet of radius $r_{k}\left(t+1\right)$ that requires higher order recursions. For example, $n_{3}\left(t+1\right)-n_{3}\left(t\right)=f_{13}n_{1}\left(t\right)n_{3}\left(t\right)+f_{22}n_{2}{\left(t\right)}^{2}+f_{23}n_{2}\left(t\right)n_{3}\left(t\right)$ is determined by three transition rates $f_{13}\left(t\right),f_{22}\left(t\right)$ and $f_{23}\left(t\right)$ and, therefore, required three successive distributions as initial conditions to solve the recursion:

(2) $$\begin{array}{ccc} n_{3}\left(t+1\right)-n_{3}\left(t\right) & = & f_{13}\left(t\right)n_{1}\left(t\right)n_{3}\left(t\right)+f_{22}\left(t\right)n_{2}{\left(t\right)}^{2}+f_{23}\left(t\right)n_{2}\left(t\right)n_{3}\left(t\right),\\ n_{3}\left(t+2\right)-n_{3}\left(t+1\right) & = & f_{13}\left(t+1\right)n_{1}\left(t+1\right)n_{3}\left(t+1\right)+f_{22}\left(t+1\right)n_{2}{\left(t+1\right)}^{2}\\ & & +f_{23}\left(t+1\right)n_{2}\left(t+1\right)n_{3}\left(t+1\right),\\ n_{3}\left(t+3\right)-n_{3}\left(t+2\right) & = & f_{13}\left(t+2\right)n_{1}\left(t+2\right)n_{3}\left(t+2\right)+f_{22}\left(t+2\right)n_{2}{\left(t+2\right)}^{2}\\ & & +f_{23}\left(t+2\right)n_{2}\left(t+2\right)n_{3}\left(t+2\right). \end{array}$$

Although in principle the transitions rates $f_{ij}\left(t\right)$ in Equation (2) could change over time to reflect the changes in thermophysical conditions (temperature quenches, external pressure, etc.), we assumed that, for the purpose of estimating them from the radii distributions at successive images, they remain quasiconstant. A possible justification for such an assumption is the fact that all our images are acquired on the thermal plateau (see Figure 1c). Additionally, even if the transition rates $f_{ij}$ change over time, we assumed that they *do not* change dramatically over, for example, three successive images. As a result, the transition rates $f_{ij}$ for a 3-pixel bin are solutions of the linear system:

(3) $$\begin{array}{c} \left(\begin{array}{c} n_{3}\left(t+1\right)-n_{3}\left(t\right)\\ n_{3}\left(t+2\right)-n_{3}\left(t+1\right)\\ n_{3}\left(t+3\right)-n_{3}\left(t+2\right) \end{array}\right)=\left(\begin{array}{ccc} n_{1}\left(t\right)n_{3}\left(t\right) & n_{2}{\left(t\right)}^{2} & n_{2}\left(t\right)n_{3}\left(t\right)\\ n_{1}\left(t+1\right)n_{3}\left(t+1\right) & n_{2}{\left(t+1\right)}^{2} & n_{2}\left(t+1\right)n_{3}\left(t+1\right)\\ n_{1}\left(t+2\right)n_{3}\left(t+2\right) & n_{2}{\left(t+2\right)}^{2} & n_{2}\left(t+2\right)n_{3}\left(t+2\right) \end{array}\right)\left(\begin{array}{c} f_{13}\\ f_{22}\\ f_{23} \end{array}\right). \end{array}$$

As we notice from Equation (1), for larger droplets, the multiplicity associated with the possible binary collisions increases quickly, which requires longer recursions.

In the WFOV images, we identified droplets at the lower end of an image resolution limit of a 1-pixel radius only in about 10% of analyzed images, which gives quite a space matrix when solving Equation (1). As a result, if we were to use the measured $n_{1}\left(t\right)$ for predicting $n_{j}\left(t+1\right)$, then the solutions of Equation (1) could not always be determined. Therefore, we dropped any contribution of $n_{1}\left(t\right)$ from all recursions shown in Equation (1).

**Figure 6:**
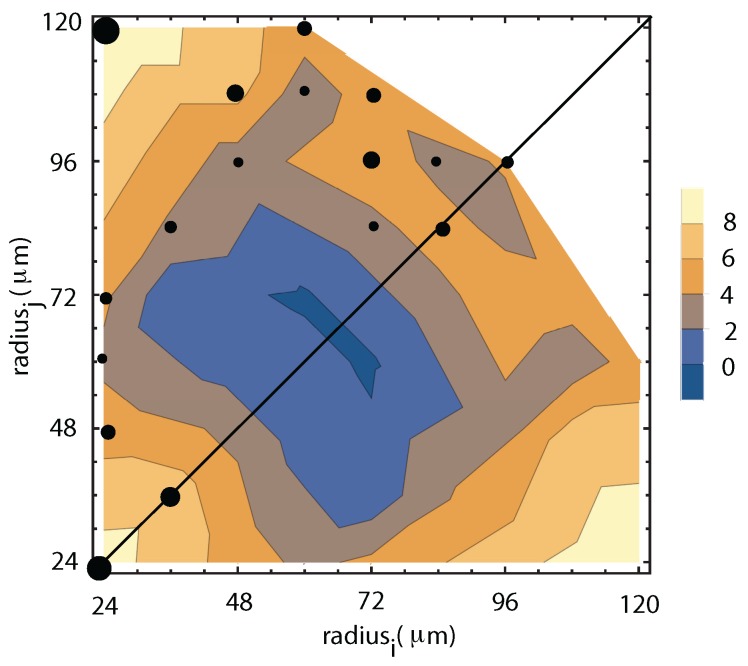
A logarithmic contour plot of the positive transition rates for WHOV images show solid circles proportional to the relative power law exponent (see the color legend). The logarithmic contour plot shows two dominant mechanisms for generating droplets: (1) small droplets that collide with droplets of any radius; and (2) preferential coalescences of large droplets with similar size.

**Figure 7:**
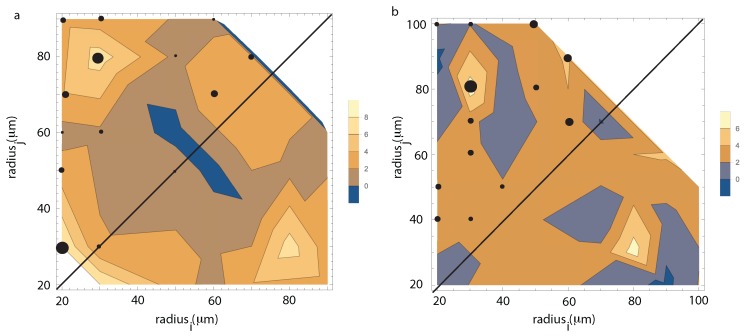
(**a**) the NFOV images of liquid droplets collisions inside the large vapor bubble. The landscape of the positive transition rates is dominated by two mechanisms: (1) low and similar radii (20 μm with 30 μm); and (2) asymmetric sizes at 30 μm and 80 μm; (**b**) NFOV images of vapor bubbles inside the liquid matrix. The transition rates for binary collisions are dominated by 30 μm and 80 μm sizes. At the same time, similar size collisions are much more likely than in the case of liquid droplets. All contour plots are shown on a logarithmic scale. The size of the solid circle is proportional to the transition probability exponent.

In general, both in the WFOV and NFOV analysis, we identified two dominant tendencies based on the clustering of the transition rates. One is an asymmetric coalescences that favors coalescences between small droplets, usually, $r_{i}\approx$ 20–30 μm and a broad range of other radii $r_{j}\in$ (30,120) μm (see Figure 6 and Figure 7). The other cluster favors almost symmetrical coalescences between droplets of similar radii such as $r_{i}\approx$ 80 μm and a broad range of other similar radii, usually, $r_{j}\in$ (60,90) μm.

The landscape of the positive transition rates $f_{ij}$ for WFOV contains different combinations of radii $r_{i}$ and $r_{j}$ (see Figure 6) with the above-mentioned two dominant clusters: one that generates collisions between small radius droplets $r_{i}\approx$ 20–30 μm and a wide range of other radii $r_{j}\in$ (20,120) μm; and another subset with a radius around $r_{i}\approx$ 70 μm that generated binary collisions with similarly large droplets with $r_{j}\in$ (80,100) μm.

For the NFOV images, we investigated separately the collision mechanism for the liquid droplets inside the large vapor bubble (see also Figure 7a) from the collision mechanism for the vapor bubbles inside the liquid matrix (see Figure 7b). As radii distributions change over time, the collision landscape of the positive transition rates $f_{ij}$ for different combinations of radii $r_{i}$ and $r_{j}$ would also change over time. Therefore, we again averaged over specific frames and plotted the contours of equal transition rates on a logarithmic scale (see Figure 7).

For the NFOV images of liquid droplet collisions inside the large vapor bubble, we found that the maximum positive transition rates (marked with solid circles in Figure 7a) have a distinctive pattern that favors large transition rates $f_{ij}$ for binary collisions between small droplets $r_{i}\approx$ 20 μm and a wide range of large droplets with $r_{j}\in$ (30,90) μm. However, as we notice from Figure 7a, there are two dominant collision processes: (1) between droplets of almost equal size of 20 μm and 30 μm; and (2) between droplets of 30 μm and 80 μm. Both collision mechanisms suggest that the droplets of small radii will rapidly disappear in favor of larger droplets. The second group of relatively highly probable collisions is around $r_{i}\approx$ 60–70 μm that produces coalescences with similarly large droplets with $r_{j}\approx$ 70–80 μm (see Figure 7a). The second highly probable mechanism leads to a fast growth of the fraction of large droplets (as seen in Figure 7b).

For the NFOV images of vapor bubbles inside the liquid matrix, we found that the largest transition rates (marked with filled circles in Figure 7b) show a significantly different behavior, i.e., the binary collisions are primarily driven by asymmetric size bubbles such as 30 μm and 80 μm (see Figure 7b). At the same time, similar size collisions, however, are more evenly distributed across all sizes compared to liquid droplets case. For example, binary collisions between vapor bubbles of 30 μm and 40 μm have almost the same transition probability as 60 μm and 70 μm collisions.

## 6. Conclusions

There are two major novelties of this study: (1) we recorded during the same thermal plateau both the evolution of liquid droplets statistics immersed in a vapor bubble and the evolution of vapor bubbles immersed in the liquid matrix; and (2) based on the respective statistics we computed the transition rates that govern the evolution of individual radii. Both the WFOV and NFOV image analysis showed that the droplet distributions could be fitted with a lognormal function and the peak of the distributions shifts towards larger droplet radii over time. By following the temporal evolution of narrow bins of droplet radii (1-pixel increment in WFOV and 10 pixels increment in NFOV), we noticed that small radii distributions reduce their contribution over time, whereas the large radii distributions contribution increase. Since we only observed binary coalescences in all images, we derived a mathematical model that describes that rate of change of droplet radii distributions. By recursively solving for the transition rates, we identified two characteristic coalescence mechanisms: (1) dominantly asymmetric coalescences between one droplet of small radius (2 pixels in WFOV) and a broad range of other radii (between 2–10 pixels in WFOV); and (2) dominantly symmetric coalescences between one droplet of large radius (eight pixels in WFOV) and similar radii (between 6–8 pixels in WFOV).

Our novel approach to extracting the transition rates from images of liquid droplets and vapor bubbles, respectively, would allow us in the future to derive a realistic phenomenological model of droplet distributions, their evolution, and their dependence on different thermophysical parameters. It is known that, for large droplet diameters and binary coalescences, the rate of change of the number of domains was estimated as $dN/dt=-N^{2}\int_{\Sigma }p\left(r\right)V\times nd\Sigma$, where *N* is the number of domains per unit volume, p(r) is the pair distribution function of the tubes/droplets, V is the relative velocity of the tubes/droplets, and n is the outward normal to the collision surface Σ [31,46]. In these references, it is found that the long tail of the droplet size distribution, such as those found in both our WFOV and NFOV images can be reasonably modeled mathematically for radii over a natural cutoff with a power law, i.e., $dN\left(r\right)/dr=Ar^{\theta }$ [31,47]. Molecular dynamic simulations showed that, for a large volume fractions of the minority phase, the distribution of droplets versus their corresponding diameters becomes wider over time (see Figure 7b in [47]). Our study opens the possibility of modeling droplet dynamics not only in the limit of large radii, but for all radii, and could offer a more complete quantitative view of coalescence processes during phase separation.
